# Supplementary material for: The Bloom syndrome complex senses RPA-coated single-stranded DNA to restart stalled replication forks
Source: Nat Commun. 2021 Jan 26;12:585. doi: 10.1038/s41467-020-20818-5 (PMC7838300; doi:10.1038/s41467-020-20818-5)
Supplement: Supplementary file 1 — Supplementary Information [file 41467_2020_20818_MOESM1_ESM.pdf]

Fig. S1a

BLM

```
                         TR(TOP3A/RMI1-binding domain)

Human        1 ---MAAVPQNNLQEQLERHSARTLNNKLSLSKPKFSGFTFKKKTSSDNNVSVT-NVSVAKTPVLRNKDVNVTEDFSFSE-
Mouse        1 MRIMAAVPLNNLQEQLQRHSARKLNNQPSLSKPKSLGFTFKKKTSEG-DVSVT-SVSVVKTPALSDKDVNVSEAFSFTES
Dog          1 ---MAAVPQNNLQEQLERHSARKLSNKLSPSKPKSSVFTFKKKTSAN-NVSVT-SVSVAKTPVLNDKDVNVTEAFPFSE-
Chicken      1 ---MAAVPQNNLREQLRLHSARGALSKVLPARSRPAAFTFRRTHSPA-----------AGPAALRDKDVNTSLGSGGVA-
Xenopus      1 ---MAALPQNNLQKQLELFPAKGTSNKLSLQKTKSSVFTFKKKCSPN-VSAST-GFIPFQQHVLKDKNVNVKQDGTHTA-
Zebrafish    1 ---MSSLPQNNLKEHLERHNN-AAQNKLSLLKPKPGGFCFKKKSSSG-ISKVEVPQKVTGSSVLANRSVNIPSNFEVTKN


Human       76 PLPNTTNQQRV---KDFFKNAPAGQETQRG--GSKSLLPDFLQTPKEVVCTTQNTPT-VKKSRDTALKKLEFSSSPDSLS
Mouse       79 PLHKPKQQAKI---EGFFKHFPGRQQSKGT--CSEPSLPATVQTAQDTLCTTPKTPT-AKKLPVAVFKKLEFSSSA---D
Dog         75 PLPHATSQQTR---PSDLKNTPAGQQTRRT--VSKPLLPDLSL-VSRVLCNTQNTPI-VKKSSNATFKKLEFSSSSDSFV
Chicken     66 ---------RP---KDLR-VGPPG---------ARPALPGPAG---------SSGPT-AGAPPGSV--SVSLPSSGPAAY
Xenopus     75 -LPKATERNKI---NCFFT--PVYTKS---------GQPPQVVALKDHVHGNDSANK-PPSTEDAASKKTGINTSFGSVT
Zebrafish   76 PVTFSQKPERVAPKVNFF-TAPSKPKTNTVNPLANPFALNKTPLVQSTIKALPKSAALPKDSTNDQTVELKHDIS---QL

                     1

Human      150 TINDWDDMDDFDTSETSKSFVTPPQSHFVRV-STAQKSKKGKRNFFKAQLYTTNTVKTDLPPPSSESEQIDLTEEQKDD-
Mouse      150 SLSDWADMDDFDMSASDA-FASLAKNPATRV-STAQKMKKTKRNFFKPPPRKANAVKTDLTPPSPECLQVDLTKESEEEE
Dog        148 TINDWDDMDDFDTSGNSK-LVTPCKNHFVRV-STAQKSKKPKRNFLKAQLPQANTVKADLTPSSFESKQACLTKKQNDD-
Chicken    112 TGDEWDDMDDFDLSGFEKKFSRP-AVLSPKG-P-----RTPQ-----GRGLRVSKARADVGPQEHGSEVSCS-Q------
Xenopus    139 SLEEWDDLDDFDTSVSPP------KSHAGKGGKTPQKCKNTS--------PVASFKIQSISPEGPTTEKHDCAKLLYDNN
Zebrafish  152 SFNEWDDLDDFETPVKSR-VASPVAGTSTKK-PSV-SDQNTS--------S----------SCSSKCEETKVNEE-SQA-


Human      228 --------------SEWLSSDVICIDDGPIAEVHINEDAQESDS-LKTH--------------LEDER-------DN---
Mouse      228 EE------EEEAEGADCLSRDVICIDNDSASEELTEKDTQESQS-LKAH--------------LGAER-------GD---
Dog        225 --------------SEWFSNDVICIDDDPNPEELIHGDPQESHS-LKTH--------------LEEER-------D----
Chicken    173 ---------------GSSQGSLICLGDAGAPAAAVLSDNGMEEARAATNG----------GSGESQKL-------SN---
Xenopus    205 EVASEPRKNLHAKTAESPDQSLVCLASVE-P----------------TN--------------L--ER-------DM---
Zebrafish  209 ------------TETITAPKDVLSAANGVST-ETAEREPEDSPI-KKSKRPKKSVQHTALLSDTEDEEIIHCVSPDTNQK

                                                        2

Human      269 -------SEKKKNLEEAEL-HSTEKVPCI-EFDDDDYDTDFVPPSPEEIISASSSSSKCLSTLKDLDTSDRKEDVLSTSK
Mouse      277 -------SEKKSHEDEAVF-HSVQNTEYF-EHNDNDYDIDFVPPSPEEIISTASSSLKCSSMLKDLDDSDKEKGILSTSE
Dog        265 -------SEK-KNWEETEL-CSLEKSPCV-ELDEDDYDTDFVPPSPEV-ISASSSSLKCFSMLKDLDASGT-EDGPSTSE
Chicken    218 -------GEKSSQLEPGDV--GNELLADI-ELEEDDY-LDVVPPSPEEELPSFSPSVRNVSNIFKESPTDGRSAVHGTE-
Xenopus    242 -------CRNTDYLGTDDLEHDQETLSQVLIEEEDDCEPDFIPPSPSDESLSSPPVLKVISAQRKHKVS--------SLT
Zebrafish  275 NFKSVAVAEK-DKWGEPN---VIDSDDCE-ENNHYEGFEDFIPPSPIPEEISLSVSDKEKSSSEPVTPANKKESSRSAS-

                                                   DHBN (dimerization domain)

Human      340 DLLSKPEKMSMQELNP--ETSTDCDARQI-SLQQQLIHVMEHICKLIDTIPDDKLKLLDCGNELLQQRNIRRKLLTEVDF
Mouse      348 ELLSKPEEMTTHKSDA--GTSKDCDAQQI-RIQQQLIHVMEHICKLVDTVPTDELEALNCGTELLQQRNIRRKLLAEAGF
Dog        333 D-LPTPKGMTTQQPNQ--ETSTNCDARQP-SLQQQLIHVMDQICKLVDTIPDDELKALTCGNALHQQRDIRRKLLAEADF
Chicken    286 ---SEPELMAPKQPAAEQDSSAEHADKGL-HLEQQLYSVMEDICKLVDAIPLHELTSISCAKELLQQRELRRKLLADSGA
Xenopus    307 D-VNDCENTTDHLQGQ--SVSTSLDS----KVPSQLLTLMLEICDLVDKIPISELHVLSCGLDLKKKRDMRKRLLSNDSV
Zebrafish  349 G-LPAPL---------------DQSAKGLKGADDALFSVMESICCLVDTIPEHELIALTCGTELLLQRAHRKRILAKGGS


Human      417 -NKSDASLLGSLWR----YRPDSL------DGPM-------------EGDSCPTGNSMKE--------------------
Mouse      425 -NGNDVRLLGSLWR----HRPDSL------DNTV-------------QGDSCPVGHPNKE--------------------
Dog        409 -NTSDASVLASVWT----RGPDSL------GSPVEDMASLSAPDVFPKGNSHPTGNSVKE--------------------
Chicken    362 LNTNSVNG-PRNWKACVQQDPSSRPGTPLCSGPG-------------RGV-SSVGSTPKS--------------------
Xenopus    380 FRSSPADS-----------------------STV-------------SLTSCTSSTQNRDFNVNAPKGAESLSGSSVSKV
Zebrafish  413 -SRTSHS------------------------------------------DSVSTPGFLNR--------------------


Human      453 LNFSHLPSNSVSPGD---------CLLTTTL-----GKTGFSATR--KNLFERPLFN------THLQK------------
Mouse      461 LNSPYLLSHSPSTEE---------CLPTTTP-----GKTGFSATP--KNLFERPLLN------SHLQK------------
Dog        458 LNFPHLPSHSSSTGE---------CLLATTP-----GKTGFSGTM--KNPSERLLFS------PHLQK------------
Chicken    407 TNLPSVLS--------------------RTV-----NSSSFSTIR--NQTLDKL--DTSYSSKETDQEVICLEPAALPSP
Xenopus    424 FKFNKLAVHDIGTKESENSANSAPNFMEKIG-----NKTSFSFRAGGDSIMEN-SFN--------FHS------------
Zebrafish  430 PTFGVTPSNL----T---------SLTPVTSGKREGVKTGFSFRK--S-IASVM--SVGD--ESVFED------------
```

Fig. S1a

BLM

```
Human      499 ----------------SFVSSNWAET---------------------------------PRL-----------------
Mouse      507 ----------------SFVSSNWAET---------------------------------PRM-----------------
Dog        504 ----------------SFVSSNWAET---------------------------------PRI-----------------
Chicken    458 KVNGKGSTSLSRPSEASFNGSWCEK----------------------------------PT------------------
Xenopus    478 ----------------SVLSNSRFNT---------------------------------PQN-----------------
Zebrafish  478 ----------------SDCIINGVETPGGTWNPNSSTKISAGRDTFNGSIQTLSKPESKTDKCYSRLSFNESSNQTDLFYS

                                                                          3
Human      512 ------GKKNESSYFPGNVLTSTAVKDQNKHTA--SINDLERETQPSYDIDNFDIDDFDDD---DDWEDIMHNLAASKSS
Mouse      520 ------ENRNESTDFPGSVLTSTTVKAQSKQAA--SGWNVERHGQASYDIDNFNIDDFDDDDDDDDWENIMHNFPASKSS
Dog        517 ------EKRNESSYFLGNVLTSTAVRDQNKRAA--PINDLE--IQAACDIDNFDIDGFDDD---DDWEDIMHDLAASRSS
Chicken    485 ------GRDSGNWRVPERPTASTALKAQHTAPAGNPASGCWDVNDTDFDLDHFDIDDFDE-----GWEEAVAPEAAPEAP
Xenopus    491 ------EKP---------ISSSTCTRP---YSQ--PIDDMD-NPDLDFDIDNFDIEDLDDI---HCLDSP-AAPSVSSKN
Zebrafish  543 PKRVDSGSRNADSSVEINIAGSSSLRT---GAE--PVD--------DFLLDDFDIDDFDENDIPDYYEEPPSVLESRNNS

                                                                                 Helicase
Human      581 -TAAYQPIKEGRPIKSVSERLSSAKTDCLPVS-STAQNINFSESIQNYTDKSAQNLASRNLKHERFQSLSFPHTKEMMKI
Mouse      592 -TATYPPIKEGGPVKSLSERISSAKAKFLPVV-STAQNTNLSESIQNCSDKLAQNLSSKNPKHEHFQSLNFPHTKEMMKI
Dog        584 -TAACLPIKEGRPVKPVSERISSAKTNCLPVA-STAQNKN-SESIQNYTDKSAQNLASRNLKHERFQSLSFPHTKEMMKI
Chicken    554 PAPQWQPLREGSA--SLRCRLLAAAAGSAPGPHPTAPKSG-----CGISAKSSSEPLVHNPAHERFRGMKFSHSEEMLKI
Xenopus    546 -VPQYPTIREAQLDSRNKEKNTRN---------NTGDTTN-----PSLLSDSLLKPQIENPAHERFRGFNFPHSKEMMKI
Zebrafish  610 -GVKTPSVQEGGSSKSFERKTF-----TPPAP-KSIK-------------TPNPEPLYRNPAHDRFRGFNFPHSPEMMKI

                                              Helicase
Human      659 FHKKFGLHNFRTNQLEAINAALLGEDCFILMPTGGGKSLCYQLPACVSPGVTVVISPLRSLIVDQVQKLTSLDIPATYLT
Mouse      670 FHKKFGLHNFRTNQLEAINAALLGEDCFILMPTGGGKSLCYQLPACVSPGVTIVISPLRSLIVDQVQKLTSFDIPATYLT
Dog        661 FHKKFGLHNFRTNQLEAINAALLGEDCFILMPTGGGKSLCYQLPACVSPGVTIVISPLRSLIVDQVQKLTSLDIPATYLT
Chicken    627 FHRKFGLHSFRTNQLEAINAALLGEDCFILMPTGGGKSLCYQLPACVSAGVTVVISPLRSLIIDQVQKLKTLDIASTYLT
Xenopus    611 FHKKFGLHRFRTNQLEAINACLCGEDCFILMPTGGGKSLCYQLPGCISPGVTIVISPLRSLIVDQVQKLTSLDIPATYLT
Zebrafish  670 FHKKFGLHQFRFNQLEAINASLLGEDTFVLMPTGGGKSLCYQLPACVSAGVTVVISPLRSLIVDQVQKLTTLDICATSLS

                                              Helicase
Human      739 GDKTDSEATNIYLQLSKKDPIIKLLYVTPEKICASNRLISTLENLYERKLLARFVIDEAHCVSQWGHDFRQDYKRMNMLR
Mouse      750 GDKTDSEAANIYLQLSKKDPIIKLLYVTPEKVCASNRLISTLENLYERKLLARFVIDEAHCVSQWGHDFRQDYKRMNMLR
Dog        741 GDKTDSEATSIYLQLSKKDPIIKLLYVTPEKVCASNRLLSTLENLYERKLLARFVIDEAHCVSQWGHDFRQDYKRMNMLR
Chicken    707 GDITDADASKTYMQLSKKDPIIKLLYVTPEKVCASNRLLSALENLYNRKLLARFVIDEAHCVSQWGHDFRKDYKRLNMLR
Xenopus    691 GDKTDAEAASIYLQLSKKDPIIKLLYVTPEKVCASTRLISTMENLYERQLLARFVIDEAHCVSQWGHDFRPDYKRLNVLR
Zebrafish  750 GDKKDSEAARIYMQLSRKDPAIKLLYATPEKVCASGRMISALQNLYERGLLARLVIDEAHCVSQWGHDFRPDYKRLHELR

                                              Helicase
Human      819 QKFPSVPVMALTATANPRVQKDILTQLKILRPQVFSMSFNRHNLKYYVLPKKPKKVAFDCLEWIRKHHPYDSGIIYCLSR
Mouse      830 QKFPSVPVMALTATANPRVQKDILTQLKILRPQVFSMSFNRHNLKYYVLPKKPKKVAFDCLEWIRKHHPYDSGIIYCLSR
Dog        821 QKFPSVPVMALTATANPRVQKDILTQLKILQPQVFSMSFNRHNLKYYVLPKKPKKVAFDCLEWIRKYHPHDSGIIYCLSR
Chicken    787 KKFHSVPMMALTATANPRVQKDIQNQLEMLKPQVFTMSFNRHNLKYDVLPKKPKKVAMDCLEWIKKYHPHDSGIIYCLSR
Xenopus    771 QKFQSVPMMALTATANPRVKKDILNQLKMTKPQIFTMSFNRDNLKYEVLPKKPKRVALDCVEWIKKHHPNDSGIIYCLSR
Zebrafish  830 RMFPNVPIMALTATATPRVQKDILNQLAMTRPQVFTMSFNRNNLKYSVLPKKPKKVDEECIQWIKKYYPRDSGIVYCLSR

                                              Helicase
Human      899 RECDTMADTLQRDGLAALAYHAGLSDSARDEVQQKWINQDGCQVICATIAFGMGIDKPDVRFVIHASLPKSVEGYYQESG
Mouse      910 RECDTMADTLQREGLAALAYHAGLSDSARDEVQHKWINQDNCQVICATIAFGMGIDKPDVRFVIHASLPKSMEGYYQESG
Dog        901 RECDTMADTLQKDGLAALAYHAGLSDSARDEVQHKWINQDGCQVICATIAFGMGIDKPDVRFVIHASLPKSVEGYYQESG
Chicken    867 HECDTTAAILQKEGLAALAYHAGLTDSNRDLVQKKWVNQEGCQVICATIAFGMGIDKPDVRYVIHASLPKSIEGYYQESG
Xenopus    851 HECDTMADTLQKEGLAALAYHAGLADSNRDYVQHKWINQDDCQVICATIAFGMGIDKPDVRYVIHASLPKSVEGYYQESG
Zebrafish  910 NDCDTLADSLQRAGIAALAYHAGLSDSDREYVQNKWINQDGCQVMCATIAFGMGIDKPDVRYVIHASLPKSVEGYYQESG

                   Helicase                                RQC
Human      979 RAGRDGEISHCLLFYTYHDVTRLKRLIMMEKDGNHHTRETHFNNLYSMVHYCENITECRRIQLLAYFGENGFNPDFCKKH
Mouse      990 RAGRDGEISHCVLFYTYHDVTRLKRLIMMEKDGNYHTKETHVNNLYSMVHYCENITECRRIQLLAYFGEKGFNPDFCKKY
Dog        981 RAGRDGEISHCLLFYTYHDVTRLKRLILMEKDGNRHTRETHFNNLYSMVHYCENITECRRIQLLAYFGESGFNPDFCKKY
Chicken    947 RAGRDGEMSHCLLFYSYSDVTRLRRLILMEKDGNSHTRQTHFNNLYSMVHYCENVVDCRRIQLLAYFGETDFNPNFCKDH
Xenopus    931 RAGRDGETSHCLLFYSYHDVTRIRRLIQMEKDGNSHTKQTHFNNLYSMVHYCENVVECRRMQLLSYFGENNFNPNFCKEH
Zebrafish  990 RAGRDGEISHCVLFYSYSDVIRIKRLIAMDKDGNQQSKATHINNLHSMVHFCENVAECRRIQLLAYFGEHTFNTSFCKEH
```

Fig. S1a

BLM

```
                                                        RQC
Human     1059 PDVSCDNCCKTKDYKTRDVTDDVKSIVRFVQEHSSSQGMRNIKHVGPSGRFTMNMLVDIFLGSKSAKIQSGIFGKGSAYS
Mouse     1070 PDVSCDNCCKTKDYKTKDVTDDVKNIIRFVQEHSSSPGTRNI---GPAGRFTLNMLVDIFLGSKSAKVKSGIFGKGTTYS
Dog       1061 PDVSCDNCCKTKDYKTRDVTDDVKNIVRFVQEHSSSQGTRIKNQLGPSGRFTMNMLVDIFLGSKSAKIQSGIFGQGSTYS
Chicken   1027 PEVICDNCSRKKDYKSRNVTDEVKSIIRFVQQHCGQVGGINGNRNTGSGRYTLNMMVDIFLGAKSAKIQSGIFGKGAAYS
Xenopus   1011 TQVACDNCLGKKNYKSRDVTDDVGNIVRFVQDNCSLVQGRGKGR-SNNTRLTLNMMVDIFLGSKSAKIQTGLFGKGAAYS
Zebrafish 1070 PEVICDNCARPNKYKSRNVTDDVKKIARFVQDNCEKVGNRYGKS-AQQNRLTLNMLVEIFLGSKSARIQTGMFGVGAAYS

                                                    RQC                          HRDC
Human     1139 RHNAERLFKKLILDKILDEDLYINANDQAIAYVMLGNKAQTVLNGNLKVDFMETENSSSVKKQKAL-VAKVSQREEMVKK
Mouse     1147 RHNAERLFKKLILDKILDEDLYINANDQPIAYVMLGTKAHSVLSGHLKVDFMETENSSSIKKQKAL-VAKVSQREEVVKK
Dog       1141 RHNAERLFKKLILDKILDEDLYINANDQPIAYVMPGNKAQTVLNGHLKVDFIETENSTSIKKQKAALVAKMSQREEVVKK
Chicken   1107 RHNVERLFRKLVLDKILDEDLYITANDQAVAYVVLGEKAQAVLNGLLQVEFHETENASAIRKQRAS-VTKMSQREEMVKK
Xenopus   1090 RHNAERLFRKLVLDRIIDEELYITFNDQAVAYVKMGERAQAVLNGFLKVDFQDTESASSIRKQKASVVTNTSQREEMVKK
Zebrafish 1149 KHNAERLFKKLVLDNVLMEDLYITNSGQAVAYISAGPKAMSVLNGCMQVEFHETESASSIRKHRASVCEKVSKREEMVKK

                                                     HRDC
Human     1218 CLGELTEVCKSLGKVFGVHYFNIFNTVTLKKLAESLSSDPEVLLQIDGVTEDKLEKYGAEVISVLQKYSEWTSPAEDSSP
Mouse     1226 CLGELTEVCKLLGKVFGVHYFNIFNTATLKKLAESLSSDPEVLLQIDGVTEDKLEKYGAEVIPVLQKYSEWTVPAEDGSP
Dog       1221 CLGELTEVCKSLGKVFGVHYFNIFNTVTLKKLAESLSSDPEVLLQIDGVTEDKLEKYGAEVIPILQKYSEWTLPADDGSP
Chicken   1186 CLGELTDTCKTLGKIFDVHYFNIFSTSTLKKIAETLSSDAEVLLQIDGVTEDKLEKYGAEIIKVMDKYSEWTTPEDAACQ
Xenopus   1170 CQAELTELCKRLGKIFGVHYFNIFNTATIRRIAESLSPEPEVLLQIDGVTEDKLDKYGAELIDVLQKYSEWTLPVEDICQ
Zebrafish 1229 CLGELNDLCKKLGKVFGTHYYNIFSTATLKKIAETLSADPEVLLQIDGVTEDKLEKYGAEFIELLQKYSEWQLPAEAQAE

                                                                   NLS
Human     1298 GISLSS-SRGPGRSAAE---ELDE-EIPVSSHYFASKTRNERKRKKMPASQRSKRRKTASSG--SKAKGGSAT-CRKISS
Mouse     1306 G------ARGAPE-DTE---EEEE-EAPVSSHYFANQTRNERKRKKMSATHKPKRRRTSYGG--FRAKGGSTT-CRKTTS
Dog       1301 RISPSS-SRSTRRSAPE---EFDE-ETSVSSHYFANKTKNERKRKRMSATQRSKRRRTGFGG--SKTKGGSTA-CRKTSS
Chicken   1266 SVDTAPGSAGTP--GSE---EEAA-DDVVTSSYFGGNANQRRKRKRLPNSGESKRKKTSSGG--SQQFYSKGA-RYRRAR
Xenopus   1250 KSGGPA-NVSARR-SNS---DHDD-ESCDKSSYFSSNNKKGPKRKNSSYFGKSKKRKTGGDGQQSRSKNGNSSYARKNST
Zebrafish 1309 SSGWIDTTRGHQN-DEDDNDDDDEGGGDVTSTYFRSNSGRGAKRKQGSYSRKPKRRKGSSGQ-NSSAKGGYSS-NWSSSR

                                                  4
Human     1370 K-TKSS-SIIGSSSAS-HTSQ-ATS-GANSKLGIMAPPKPI--NRPFLKPSYAFS
Mouse     1372 K-SKFY-GVTGSRSAS-CASQ-ATS-SASRKLGIMAPPKPV--NRTFLRPSYAFS
Dog       1373 K-SKSS-NIFGPHSSL-HGSQ-AAS-GAARKLGIMAPPKPI--NRPFLKPSYAFS
Chicken   1337 R-------APGSRAAAPAQSS-ALR-GAGARLGIMAPPKPS--SRHFLQPSYAVL
Xenopus   1324 A--K--------TSSS-YISG-SKT-GADKRPGFMAPPMPQ-PNRRFLKPSYSMF
Zebrafish 1386 GGGRGGGYRGGSRGAG-RGSRSAPSGSAAKRPGFMSLPTPQAAARPFLKPSFSHL
```

RMI1 **Fig. S1b**

```
                3H                                                      OB1
Human        1 MNVT-SIALRAETWLLAAWHVKVPPMWLEACINWIQEENNNVNLSQAQMNKQVFEQWLLTDLRDLEHPLLPDGILEIPKG
Mouse        1 MSVA-SAVLRVETWLLATWHVKVPPMWLEACVNWIQEENNNATLSQAQINKQVLEQWLLTDLRDLEHPLLPDDILELPKG
Dog          1 MNVT-NIALRVETWLLATWHVKVPLTWLEACVNWVQEENDNVNLSQAQMNKQVFEQWLLTDLRDLEHPLLPDGILEVPKG
Chicken      1 MSTP-NVTARVETWLSSTWHVKVPLTWLEACINWIQEENGGGNLSQAQISRQVFEQWLLTDLRDLEYPILPDGILDAPKG
Xenopus      1 MMATSGIANRVKTWLISTWHVKVPDPWLEACINWIQEENG-SSLLQAEINKQVFEQWLLTDLRDLEFPVLPANITDSLKC
Zebrafish    1 MSVG-EVQ-VIQAWLRSEWHIQVPPAWLDACVNWIKEEADRASIPQSQLNQRVLEQWLLTDLRDLAHPVLPERISEAQKT

                                                  OB1
Human       80 ELNGFYALQINSLVDVSQPAYSQIQKLRGKNTTNDLVTAEAQVTPKPWEAKPSRMLMLQLTDGIVQIQGMEYQPIPILHS
Mouse       80 ELNGFYALQINSLVDVSQPAYSQIQKLRGKNTTNDLVSAETQSTPKPWEVRPSRMLMLQLTDGVTHIQGMEYQSIPALHS
Dog         80 ELTGFFALQINSLVDVSQPAYSQIQKLRGKNTTNDLITAETQVTPKPWEAKPSRMLMLQLTDGIVQIQGMEYQSIPVLHS
Chicken     80 ELSGFYSLQIDSLLDVSQPAYSQLQKIRGKSTVNEEVTANTQAFQKPWEAKPTRMLMLQLTDGIHQIQGMEYQPVPVLHS
Xenopus     80 ELNGFHAVQMDSLVDISLPAYSQLQKLKGKDSTNEQVTCTTQQSQKPWEAKPTRMLMLQLTDGTQHIQAMEYRPIQALNA
Zebrafish   79 VLSNRCCVQMDSLLDVSQPAYNQLQRIRGTDCSNDQVSAVTQETQRPWEAKPTRMLMLQLTDGVQNLEGMEYRPIPALNA

                                    OB1
Human      160 DLPPGTKILIYGNISFRLGVLLLKPENVKVLGGEVDALLEEYAQEKVLARLIGEPDLVVSVIPNNSNENIPRVTDVLDPA
Mouse      160 GLPPGTKILVRGCILFRLGVLLLKPENVKVLGGEVDGLSEENAQEKVLARLIGELDPTVPVIPNNSIHNVPKVSGGLDAV
Dog        160 DLPPGTKILIYGNISFRLGVLLLKPENVKLLGGEVDALLEEYAQEKVLARLIGEPDPIVSVIPNNPNQSIPGITDVLDPA
Chicken    160 NLPPGTKITVHGNVAYRLGVLLLKPENVKLLGGEVDALLEEYSQERVLARLIGEAENPDSVRQPGHEQIVPGPVDELGQT
Xenopus    160 NLSPGTKMVLQGTIVCRLGVLLLKPENVKVLGGEVEALVAEYTQTKVLSRLIGVEDNTVAQHSNVQEHAAGVAAGELGQA
Zebrafish  159 NLPPGTKLQLVGPIAVRLGVLLLKAENIKVLGGEVEQLLEIHSQSRVLCGTLGLPEETH---------------------

                     5
Human      240 LGPSDEELLASLDENDELTANNDTSSER-CFTTGSSSN-------TIPTRQSSFE-------------------------
Mouse      240 LGPSDEELLASLDESEESAANNDVAMERSCFSTGTSSN-------TTPTNPSGFE-------------------------
Dog        240 LGPSDEELLASLDENE-FAAINDTSLDRRFFSTDSSSN-------TITTRQSSFE-------------------------
Chicken    240 LGPSDEELLASLDENNDFILNNETSLESGYCSRSNNFSATSCSLTTYNENTTGFPLQ-----------------------
Xenopus    240 LGPSDEDLLASLEENEEFSINNGVPSESGYYSRNENSS-------ISSSTQHQTQDSIVRCWVDDDILEFSINNGVPSES
Zebrafish  223 PQQEPDGLVASVADSGYSSLASEASLRH--------PQ-------SQPLPQISRQ-------------------------

                                                                      6
Human      287 ------PEFVISPRPKEEPSNLS-----------------IHVMDGELDDFSLEEALLLEETVQKEQMETKELQPLTFNR
Mouse      288 ------PGCNISSRPKEKPPNQP-----------------THFTDGEFDDFSLEEALLLEETVQKEQMETKASQPLTLKE
Dog        287 ------EQRIISPKPKEKPPDPS-----------------MCFTDGELDDFSLEEALLLEETVQKEQMETEELQPLTLNR
Chicken    297 -----QESVNLLPRSDEQTP-PS-----------------VEYNDAFLNDFPLEDDILLEEEIQRELEEVP---SVARNG
Xenopus    313 GYYSRNENSSISSSTQHQTQDSIVRQTSFPMLDQTVVHVNVANNHAIEEQFDIDDELFLEEEIQRELEEMSMSQAEV---
Zebrafish  258 ------DDWDMDEIPDDDFRSIP-----------------DHFDDLPQNNNPLEDVPEDFDDIP--LDELD---NVMCPI

Human      344 NADRSI-------------------------ERFSHNPNTTNNFSLTC--KNGNNNWSEKN--------------VSEQM
Mouse      345 NTGKCM-------------------------EIFSHKPSSLNHTALIH--KQGNSNFDEKT---------------SEQM
Dog        344 TPDESIEIVSQMETEELQPLTLNRTPDESTIERVSHKPNTLGNFSLTC--KSRNNNWNKKN--------------LSEQM
Chicken    351 NVNLST-------------------------ERIPHTYRSSCGSSLNETSEKDDGCGSEKPVEVTSKQKNLARTVSTGDG
Xenopus    390 --------------------------------------------------------------------------------
Zebrafish  310 DVEVSA-------------------------GTLEDPERNKHDSS-----------------------------------

Human      383 TNEDKSFGCPSVRDQNRSIFSVHCNVPLAHDFTNKEKNLETDNKIKQT-SSSDSHSLNNKIL--NREVVNYVQKRNSQIS
Mouse      383 IHEDKFFDCASTRNHHKR-F-------SAHDFTNDSKISEVDDAAQQTLSSSNVHCLRNKIL--NRKLD--LSEKSSQIS
Dog        408 INEGKSFCSPSAGDQNSSVFSVHNNVPLPHDFTNK--DSETDNKIKQTFSSIDGHSLNNKISISNGELVNYVPKSSSHIS
Chicken    406 TSTGGSLQCNSVSQACSSAIVLLK---NPHEERRN--DLGPDESSCQSRHAPDSRQLNKD------------PVSSSKTD
Xenopus    390 ----------------------------------------------------------------------------THGS
Zebrafish  330 -------------------------------LLNGSKPEEVS--------FPSRSRLGNT-------------YQSRTLN

                                                           OB2
Human      460 NENDCNLQSCSLRSSENSINLSIAMDLYSPPFVYLSVLMA--SKPKEVTTVKVKAFIVTLTGNLSSSGGIWSITAKVSDG
Mouse      451 KENGHPFQACSSRSFENNTYLSIGMDLHSPPFIYLSVLMA--RKPKEVTTVTVKAFIVTLTGNLSSSGGFWGVTAKVSDG
Dog        486 NENDHHLQTCS-RSSEYSTPLSSAMDLYSPPFIYLSILMA--SRPKKITTVKVKAFIVTLTGNLSSSGGIWSITAKISDG
Chicken    469 PEADQQAILCR-P------GNACDLDLDSPPFTYTSLLLA--KKPETITLVKVKCFIVTLTGNLTSSNGSWGIKAKVSDG
Xenopus    394 TDLPTTLSATS-HASSAS-CTSDQNNIDIPPYTYLSTILA--SKSRSITCVKLKSFIVTLNGNLSHSCGLWNIKAKISDG
Zebrafish  358 KVPNTSLASCSTST-------LDSSACKETP-MYLCSLQAGCWPPKSPQVFRLQAFIVTLVGNLRISGGVWKIGATISDG
```

Fig. S1b

RMI1

```
                                               OB2
Human      538 TAYLDVDFVDEILTSLIGFSVPEMKQSKKDPLQYQKFLEGLQKCQRDLIDLCCLMTISFNPSLSKAMVLALQDVNMEHLE
Mouse      529 TAYLDVDFIDEILTSMIGYSVPEMKQLRKDPLKYKTFLEGLQKCQRDLIDLCCLMTISYDPSSCKGVVLELQDVGMEHVE
Dog        563 TAYLDVDFADEILTSLIGFSVPEMKQSKKDPLQYQKFLDGLQKCQRDLIDLCCLMTISFNPSLSKAMVLALQDVKTEHLE
Chicken    540 SAYLEVDFADDILTSLIGFSVPEMHTLRKDPALQPKLKDGLERCQKQLIDLCCLMTIEFNPFQSKATVLILQDTDARHLE
Xenopus    470 TGYLNVEFSDDVLTELIGFTVPEMKTLKKDPSQQSTLMEGLQKCQRMLTDFCGVMTVSYNPAKEEAVVLSLQDVTEEIMQ
Zebrafish  430 SGYLDVDLSDNMLSELIGFSAAETRVLRKDLARRGEVDSGIQHCQRELVDMCCMMSVQVDLT-GRGVVLSASPISDRECS

                   OB2
Human      618 NLKKRLN---K
Mouse      609 NLKKRLN---K
Dog        643 NLKKRLN---K
Chicken    620 QLKKRLN---K
Xenopus    550 SLQKLLR---S
Zebrafish  509 EMQKRVKEARR
```

Fig. S1c

RMI2

```
                                                OB3
Human       1 MAAAADS-FS---------GGPAGVRLPRSPPLKVLAEQLRRDAE----GGPGAWRLS-RAAAGRGPLDLAAVWMQGRVV
Mouse       1 MAAAASESLS--------SGGPGAVRLPRLPPLKVLAGQLRRHAE----GGPGAWRLS-RAAVGRAPLELVAVWMQGTVL
Dog         1 MAAAAAP-VGAAGSVAGGGGGPVAVRLPRSPPLKVLAEQLRRDAE----GGPGAWRLS-RAATGRGPLELVTVWMQGTVV
Chicken     1 MAGEA------------------------ASPPLKVLAAQLRAAGR----GAGGTWRLS-RTETGRAPLCLRAVWMQGTVL
Xenopus     1 MATRCET-SA--------SGG----FQFSSPPVKMLAGKLRECTKRS-GATPSFWLEW-GPSGGGGPLDVSLVWLQGNII
Zebrafish   1 MMGASFL-SA---------------DRARSPPVKVLSSQLREASESQSSGGKAEVVIRRLGASGRRSLQVSVVWMQGTVL

                                                OB3
Human      66 MAD---RGEARLRDPSGDFSVRGLERVPRGRPCLVPGKYVMVMGVVQACSPEPCLQAVKMTDLSDN-PIHESMWELEVED
Mouse      68 AAE---GGQARLRDSSGAFSVRGLERVPRGRPCLLPGKYVMVMGVVQACSPEPCLQAVKMTDLSDN-PVHESMWELEVED
Dog        75 TAG---GGEARLRDPSGAFSVRGLERVPRGRPCLVPGKYVMVMGVVQVCSPEPCLQAVKMTDLSDN-PVHENMWELEVED
Chicken    53 QVERGGGGSARLRDGSGHFTVLGVEDVPRGRPCLSAGKYVMVMGMVRACSPEPVLRAIKMTDLSEN-PVHEEMWGLEVEE
Xenopus    66 QVRPDRGTTLRLDDTTHTFTVCGADKVPKGKPCLEIGKYVMVMGVVLSSSPEPILRAVKITDLSDN-PVHSLMWKTEVDH
Zebrafish  65 ELQAD-NNTLLMLDETGNFTVNGINSVPKGKPCLSPGKYVMVMGVIQSHSPEPVLRAVKMADLSANAAIHRRNWIYEVED

                OB3
Human     142 LHRNI--P
Mouse     144 LHRNI--P
Dog       151 LHRII--P
Chicken   132 LHRVI--P
Xenopus   145 LHRNILRP
Zebrafish 144 LQRVL--P
```

Fig. S1d

TOP3A

```
                                                  TOPRIM
Human        1 MIFPVARYA---LRWLRRP-EDRAFS-RAAMEMALR-GVRKVLCVAEKNDAAKGIADLLSNGRMRRREGLSKFNKIYEFD
Mouse        1 MIFPVTLLA---FQWHRRP-GGRALS-RAAMEVAFR-GVRKVLCVAEKNDAAKGIADLLSNGRMRRKEGLSKFNKIYEFD
Dog          1 MIFPVARSA---LRWLLRP-GARTLS-RAAMEVAFR-GVRKILCVAEKNDAAKGIADLLSNGRMRRREGFSKFNKIYEFD
Chicken      1 MNFQARLLASCGVRMLPQP-W-RGFS-RAAEGPAVQ-RIRKVLCVAEKNDAARGIADLLSNSRMRRREGFSKFNKIYEYD
Xenopus      1 MVILSRVLALSKFKVVLRP-AVRFCSHRTAAEMALRIPVQKVLCVAEKNDAAKGIADLMSNSRMRRREGFSKFNKIYEYE
Zebrafish    1 MNVLFR--------ALRKTVNSRFYR-HISVSMIRRTQIKKVLCVAEKNDAAKGIAEIMSNGRSRRREGCSVYNKIYEYE

                                       TOPRIM
Human       75 YHLYGQNVTMVMTSVSGHLLAHDFQMQFRKWQSCNPLVLFEAEIEKYCPENFVDIKKTLERETRQCQALVIWTDCDREGE
Mouse       75 YHLYGQNVTMIMTSVSGHLLAHDFQMQFRKWQSCNPLVLFEAEIEKYCPENFIDIKKTLERETHHCQALVIWTDCDREGE
Dog         75 YHLYGQNVTMVMTSVSGHLLAHDFQMQFRKWQSCNPLVLFEAEIEKYCPENFVGIKKTLQRETQQCQALVIWTDCDREGE
Chicken     77 YQMFGQNVTMVMTSVSGHLLAHDFKLPFRKWHSCNPLALFDAEIEKYCPENYVDIKRTLEREVQQCQALVIWTDCDREGE
Xenopus     80 YHLFGKDVTVVMTSVSGHLLALEFMDHMKKWHSCNPVSLFEAEVQKYCPDDFVNIKRTLEREVRQCQALIIWTDCDREGE
Zebrafish   72 YNLFGQNVTVNMTSVSGHLLALEFKAPFQKWHSCSPVLLFDAEVEKYCPENFIPIKRTLEREVRQCQALIVWTDCDREGE

                 TOPRIM                      TopA
Human      155 NIGFEIIHVCKAVKPNLQVLRARFSEITPHAVRTACENLTEPDQRVSDAVDVRQELDLRIGAAFTRFQTLRLQRIFPEVL
Mouse      155 NIGFEIIHVCKAVKPNLRVLRARFSEITPHAVRTACENLTEPDQRVSDAVDVRQELDLRIGAAFTRFQTLRLQRIFPEVL
Dog        155 NIGFEIIHVCRAVKPNLQVLRARFSEITPRAVRVACENLTEPDQRVSDAVDVRQELDLRIGAAFTRFQTLRLQKIFPEVL
Chicken    157 NIGFEIIHVCKAVKPNLQVFRARFSEITLHAVRRACENLTQPDQKTSDAVEVRQELDLRIGAAFTRFQTLRLQKIFPDIL
Xenopus    160 NIGFEIIHVCKSVKPNLQVFRARFSEITPRSIRMACEHLVQPDQNINDAVDVRTELDLRIGAAFTRFQTLRLQKIFPSVL
Zebrafish  152 NIGFEIIDVCKAVKPNIQVFRARFSEITPNSIRRACETLTEPDINVSDAVDVRQELDLRIGASFTRFQTLRLQKIFPESL

                                       TopA
Human      235 AEQLISYGSCQFPTLGFVVERFKAIQAFVPEIFHRIKVTHD-HKDGIVEFNWKRHRLFNHTACLVLYQLCVEDPMATVVE
Mouse      235 AEQLISYGSCQFPTLGFVVERFKAIQAFVPEVFHKIKVTHD-HKDGTVEFNWKRYRLFNHTACLVLYQLCMEDPMATVVE
Dog        235 AEQLISYGSCQFPTLGFVVERFKAIQAFVPEIFHKIKVTHD-HKDGIVEFNWKRHRLFNHTACLVLYQLCMEDPTATVVE
Chicken    237 ADQLISYGSCQFPTLGFVVERFKAIQAFVPEAFYKIKVTHD-HEDGNVVFNWKRNRLFNHTACLVLYHMCMEDPIATVVE
Xenopus    240 SNQLISYGSCQFPTLGFVVERFKAIQAFIPETFYKIKVTHE-HEDGLVEFSWKRNRLFNHTACLVLYQICMEDPMATVME
Zebrafish  232 SDQLISYGSCQFPTLGFVVERFKAIQAFIPETFFKIKVVHEPNEEESVEFNWKRHRLFNHTACLVLYQMCMEEPMAKVIS

                                       TopA
Human      314 VRSKPKSKWRPQALDTVELEKLASRKLRINAKETMRIAEKLYTQGYISYPRTETNIFPRDLNLTVLVEQQTPDPRWGAFA
Mouse      314 VRSKPKSKWRPQALDTVELEKLASRKLRINAKETMRIAEKLYTQGYISYPRTETNIFPKDLNLVALVEQQTVDPHWGAFA
Dog        314 VRSKSKSKWRPQALDTVELEKLASRKLRINAKETMRIAEKLYTQGYISYPRTETNIFPKDLDLTVLVEQQTPDPRWGAFA
Chicken    316 VGSKPKSKWRPLPLDTVELEKLASRKLKINAKETMRIAEKLYTQGFISYPRTETNIFPKELNLSALVQHQTQDPNWGAFA
Xenopus    319 VGSKPKSKWRPVALDTVELEKLASRKLKIGAKETMKTAEKLYTQGFISYPRTETNIFPKDLNLTSLVEQQTHDPQWGGFA
Zebrafish  312 VTSKPKSKWRPLPLDTVELEKLASRKLRISAKETMKIAEKLYTQGFISYPRTETNMFPQNLNLTRLVEQQTQDQEWGNFA

                                       TopA
Human      394 QSILERGGPTPRNGNKSDQAHPPIHPTKYTNNLQGDEQRLYEFIVRHFLACCSQDAQGQETTVEIDIAQERFVAHGLMIL
Mouse      394 QTILERGGPTPRNGSKSDQAHPPIHPTKYTSGLQGDDRRLYEFIVRHFLACCSQDAQGQETTVEIDIAQERFVAHGLIIL
Dog        394 QSILERGGPTPRNGNKSDQAHPPIHPTKYTSSLQGDEQRLYEFIVRHFLACCSQDAQGQETTVEIDIAQERFVAHGLMIL
Chicken    396 QRILDQGGPTPRNGTKSDQAHPPIHPTKYTANLQGNEQRLYEFIVRHFLACCSQDAKGQETTVEIDIANERFVAQGLMIL
Xenopus    399 QRILERGGPTPRNGNKTDQAHPPIHPTKYTNNLQGNEQRIYEFIVRHFLACCSQDAQGQETTVEIDIAAERFVAHGLMII
Zebrafish  392 QRILESGGPTPRNGNKSDQAHPPIHPTKYTNGLQGNEKRLYEFIVRHFLACCSKDAQGQEVTVEIDIAEERFSASGLTII

                                       TopA
Human      474 ARNYLDVYPYDHWSDKILPVYEQGSHFQPSTVEMVDGETSPPKLLTEADLIALMEKHGIGTDATHAEHIETIKARMYVGL
Mouse      474 ARNYLDVYPYDHWSDKLLPVYEQGSHFQPSTVEMVDGETSPPQLLTEADLIALMEKHGIGTDATHAEHIETIKARMYVGL
Dog        474 ARNYLDVYPYDRWSDKTLPVYERGSHFQPSTVEMVDGETSPPQLLTEADLIALMEKHGIGTDATHAEHIETIKARMYVGL
Chicken    476 ARNYLEVYPYEKWSDKVIPLYQKGSRFQPTTVEMVDGETSPPLLLTEADLIALMEKHGIGTDATHAEHIETIKTRMYVGL
Xenopus    479 ARNYLDVYPYDKWNTKIIPVYEMGAQFQPTTVEMVDGETSPPQLLTEADLIALMEKHGIGTDATHAEHIETIKSRSYVGL
Zebrafish  472 ARNYLEVYPYDKWYNKVIPSYSPNTTFQPTAIEMVEGQTSPPQLLTEADLISLMEKHGIGTDATHAEHIETIKSRMYVGL

                                       TopA
Human      554 TPDKRFLPGHLGMGLVEGYDSMGYEMSKPDLRAELEADLKLICDGKKDKFVVLRQQVQKYKQVFIEAVAKAKKLDEALAQ
Mouse      554 TSDKRFLPGHLGMGLVEGYDSMGYEMSKPDLRAELEADLKLICEGKKDKFQVLRQQVQKYKQVFIEAVAKAKKLDEALSQ
Dog        554 TADKRFLPGHLGMGLVEGYDSMGYEMSKPDLRAELEADLKLICEGKKDKSVVLRQQVQKYKQVFIEAVAKAKKLDEALSQ
Chicken    556 TADQRFLPGHLGMGLVEGYDSMGYEMSKPDLRAELEADLKLICEGKKDKSIVLHQQVQKYKQVFIEAVARANKLDQALAQ
Xenopus    559 TPEQRFLPGELGMGLVEGYDSMGFQMSKPDLRAELESDLKLICEGKKDKFTVLRLQIQKYKQVFVEAAAKATKLDEALSQ
Zebrafish  552 TADQRFLPGELGMGLVEGYNSMGYEMSKPDLRAELEADLKLVSEGRKNKASVLSYHVAKYKAVFIESVRKAKKLDEALSN

                                    Zinc finger
Human      634 YFGNGTELAQQEDIYPAMPEPIRKCPQCNKDMVLKTKKN-GGFYLSCMGFPECRSAVWLPDSVLEASRDSSVCPVCQPHP
Mouse      634 YLGERTEMAQQEEIYPAMPEPVRKCPQCNKDMVLKTKKS-GGFYLSCMGFPECRSAVWFPDSVLEASRDNSVCSVCQPPP
Dog        634 YFGEGTEVAQQEAIYPSMPEPIKKCPQCNKDMVLKTKKN-GGFYLSCTGFPECRSAVWFPDSVLEASRDDSVCPVCQPHP
Chicken    636 YFGEATEIVEQEEVYPAMPVSIRKCPQCNNDMVLKTKKN-GGFYLSCTGYPACKTAVWFPDFVLDVAKDESICAVCKPHP
Xenopus    639 YFGQAAEAVPQQEIYAETPLPVRKCPQCNRDMVLKTKRD-GGFFLSCVGYPSCKSSVWFPSSVLEVSRDESICSTCRPHP
Zebrafish  632 YLGPAQEIAEQEQVELEIPLPVRKCPQCNRDMVLKKKKDSTGMLLSCLGYPACKAAVWFPDTVLEVSRDESICPTCRPHP
```

TOP3A

```
                             Zinc finger
Human       713 VYRLKLKFKRGSLPPTMPLEFVCCIGGCDDTLREILDLRFSGGPPRAS----QPSGRLQANQSL-NRMDNSQHP---QPA
Mouse       713 VYRLKLKFKRGSLPPAMPLEFVGCIGGCDETLKEIFGLRFPRALPRAS----QPSGHLQASQAL-NRMDSSQHNLS-QPL
Dog         713 VYRLKLKFKRGSLPPTMPLEFVGCIGGCDETLREILDLRFSRGPPRASQPASQPSNYLQASQSQ-NRTDSAPR-------
Chicken     715 VHRLKFKFKRGSVPPTMPLDFVGCIGGCDQMLKEILDLRYSHRSSQSGQSASQQANQLQVNNSF-NRANSENRRVG-G-T
Xenopus     718 VHKLKLKFKRGSIPPLMPLEFVGCIGGCDETLREILDLKYLHGGNRPPQAAPRESNHLQVNQTS-NRMGNSGDGRGRQPQ
Zebrafish   712 VHMLKFKFKRGSLPPMMPLEFVGCIGGCDDTLREVLNLKYLRGRDAES--APQANNSSRSSQHHIHRTSSAPPP-S-R-V

                                                         Zinc finger
Human       785 DSR--QTGSSK--ALAQT-LP-PP-TAAGESNSVTCNCGQEAVLLTVRKEGPNRGRQFFKCNGGSCNFFLWADSPNPGAG
Mouse       787 VNR--HTRPSK--TVAQA-LL-PP-TTAGESNSVTCNCGREAVLLTVRKQGPNQGRHFYKCSNGDCNFFLWADSSHSTGG
Dog         785 -----------------M-LPLPA-AADGESNSVICNCGQEAVLLTVRKEGPNQGRQFYKCGGGGCDFFLWADSSALEPR
Chicken     792 TNM--ATGHLLYLSSTRAPRPAPSAAPDDGNNAVVCNCGNEAMLLTVRKEGPNQGRQFYKCSANTCNFFLWSDQQSEDRS
Xenopus     797 TKTLKPPVVKP--STSRG-AP-PI-PGNSESNEVVCNCGVAAVQLTVRKEGPNQGRPFYKCNGGACNFFLWADQEDGGGQ
Zebrafish   787 ENI-RPPAPRP--RADPG-LQ-PP-P-STQGGAIVCNCGQDALLLTVRKEGPNQGRQFYKCNTGDCKFFLWADQPPEQ--

                                                                           Zinc finger
Human       858 GP------PALA-YRPLGASLGCP-------PGPGIHLGGFGNPGD-GSGSGTSCLCSQPSVTRTVQKDGPNKGRQFHTC
Mouse       860 GT------PTSA-SGPPGSSVGCP-------SSVGSHMDGFGSLGS-DSDGGTPCLCGQPAVTRTVQKDGPNKGRQFHTC
Dog         846 GP------PASA-LGPAGGSLGHP-------PAMGDRRGGFGRPGD-DGGRGMACLCSQPAVTRTVQKDGPNKGRQFHTC
Chicken     870 NAAPRGSAPPRA-FGARGPANSQR-------PGGKRGPEHFGSSNNSSSGDSTVCRCDQPAVTRTVQKDGPNKGRQFHTC
Xenopus     872 NA------PAGPSTGRPSNAFNNS-------RAPDSFRSGGGSNDPGSEGQAVVCMCNQPAVTRTVQKDGANKGRQFHTC
Zebrafish   858 GV------PERN-RGPLSQNVQPPRPSNGFGNVSQQRQNHRGSGG--DE-GETMCNCNEPSVTRTVMKEGPNKGRMFHTC

                  Zinc finger                                                   Zinc knuckle
Human       923 AKPREQQCGFFQWVDENTAPGTSGAPS-------WTGDRGRTLESEARSKRPRASSSDMGSTAKKPRKCSLCHQPGHTRP
Mouse       925 AKPREQQCGFFQWVDENVAPGSFAAPA-------WPGGRGKAQRPEAASKRPRAGSSDAGSTVKKPRKCSLCHQPGHTRT
Dog         911 AKPREQQCGFFQWVDENVAPGTLRTPT-------WPRGRGRTQGPEARSKRARASSADMGSTPKKPRKCSLCHQPGHTRP
Chicken     942 SKPREQQCGFFQWADENVAPGASGDFS-------ENHFGSSEYSRELGSKAKRPSNLSHGATAKKPRTCSICHQPGHTKK
Xenopus     939 SKPREQQCGYFQWADENVPPGSSSGFSGSSGGSSFSGGSGA--QRNSGKKNKRSENSFSGPTAKKPRSCGICHQPGHNRT
Zebrafish   928 GKPRDQQCGFFQWANENVAPATA-----------FSGGNGG---KPSGNKK-ASTTSVKPPTAKRQRTCGICHEPGHTRV

Human       996 FCPQNR
Mouse       998 FCPQNR
Dog         984 FCPQNS
Chicken    1015 TCPLDH
Xenopus    1017 NCPQNR
Zebrafish   993 TCPQAH
```

Fig. S1. Sequence alignments of (a) BLM, (b) RMI1, (c) RMI2 and (d) TOP3A orthologs from representative vertebrates. Underlined regions in red denote conserved protein domains/motifs. Numbered motifs in BLM and RMI1 correspond to sequences synthesized as biotinylated peptides (see main text for further details).

Fig. S2

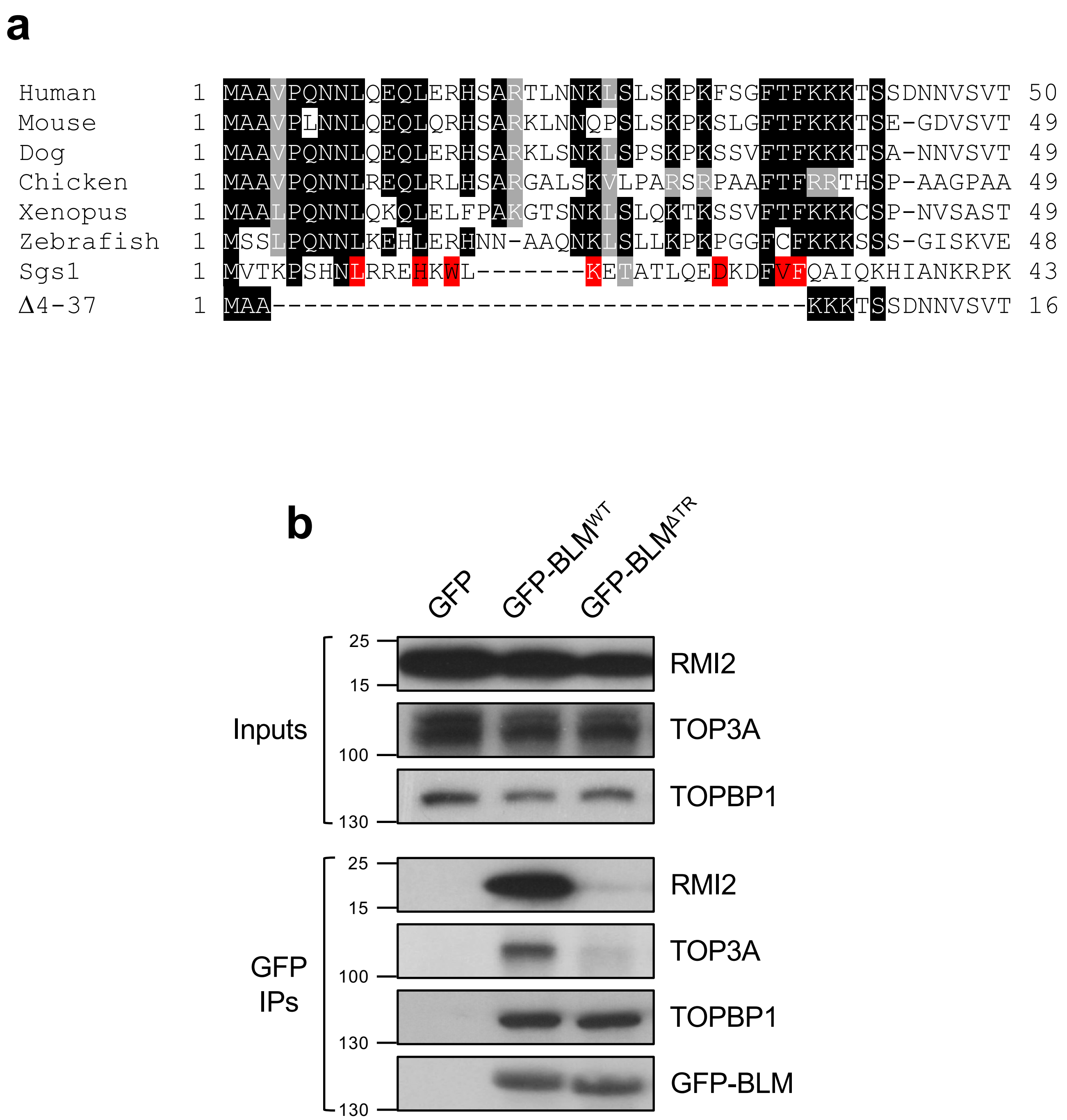

Fig. S2. Identification of a critical region required for BLM to associate with other BTR complex components.

a) Sequence alignment of the N-terminal TR region of vertebrate BLM orthologs and Sgs1. Residues in red indicate those required for Sgs1 to promote resistance to HU[31].

b) Deletion of residues 4-37 in the TR domain of BLM abrogates interaction with the BTR complex. 293FT cells were transfected with the indicated constructs and GFP-pulldowns were carried out from cell lysates, followed by western blotting for the indicated proteins.

Fig. S3

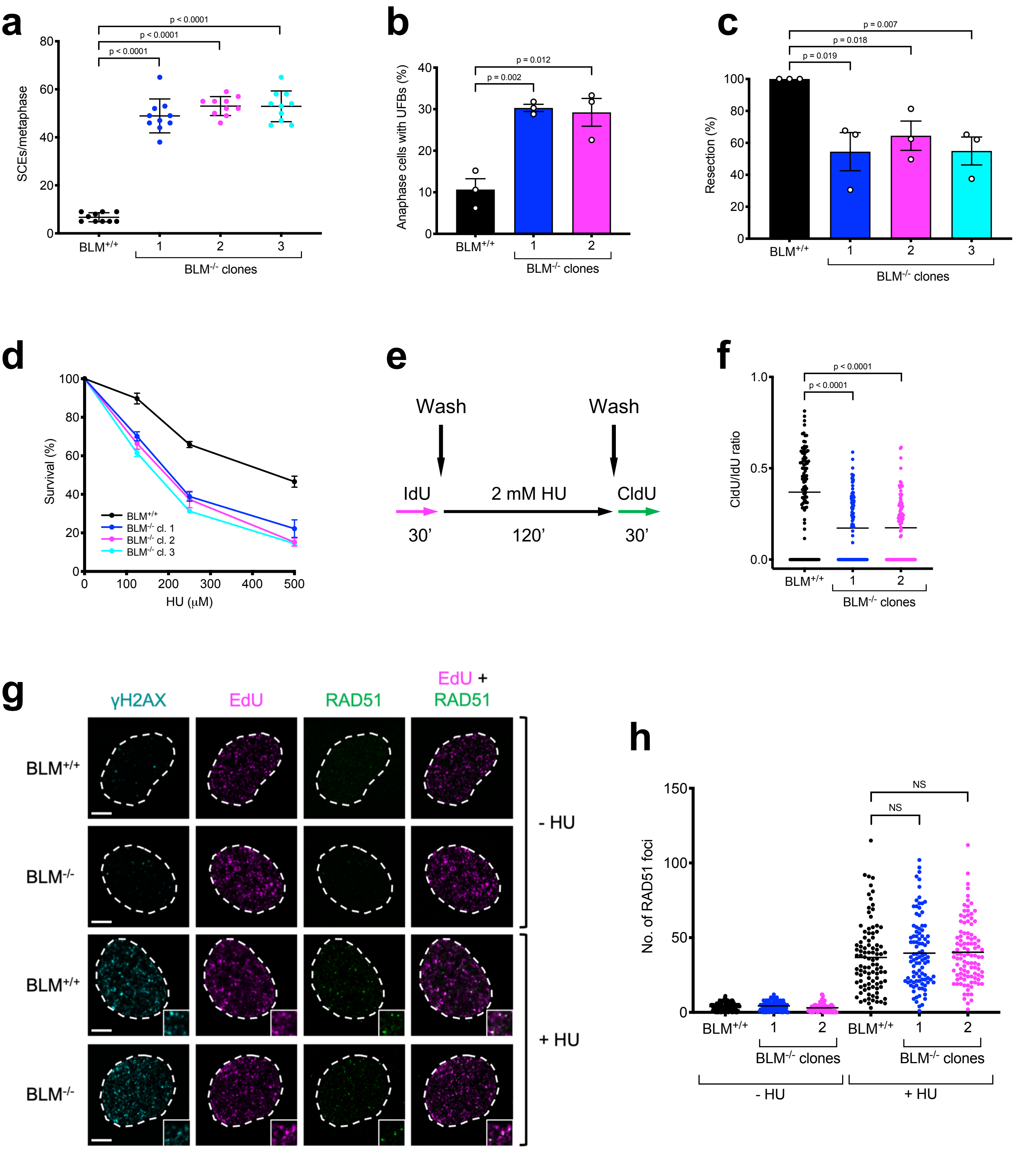

a
p < 0.0001
p < 0.0001
p < 0.0001
SCEs/metaphase
BLM+/+
1
2
3
BLM-/- clones
b
p = 0.012
p = 0.002
Anaphase cells with UFBs (%)
BLM+/+
1
2
BLM-/- clones
c
p = 0.007
p = 0.018
p = 0.019
Resection (%)
BLM+/+
1
2
3
BLM-/- clones
d
Survival (%)
BLM+/+
BLM-/- cl. 1
BLM-/- cl. 2
BLM-/- cl. 3
HU (μM)
e
Wash
Wash
IdU
2 mM HU
CldU
30'
120'
30'
f
p < 0.0001
p < 0.0001
CldU/IdU ratio
BLM+/+
1
2
BLM-/- clones
g
γH2AX
EdU
RAD51
EdU +
RAD51
BLM+/+
BLM-/-
BLM+/+
BLM-/-
- HU
+ HU
h
NS
NS
No. of RAD51 foci
BLM+/+
1
2
BLM+/+
1
2
BLM-/- clones
BLM-/- clones
- HU
+ HU

Fig. S3. Characterization of *BLM*$^{-/-}$ RPE-1 cells.

a) SCE assay showing that all three *BLM*$^{-/-}$ clones have very high levels of SCEs (n=3, one representative experiment shown). At least 10 metaphases were scored per sample. Error bars denote SD. Significance was determined using the two-sided Mann-Whitney *U* test.

b) Quantification of the increase in UFBs observed in *BLM*$^{-/-}$ cells by immunofluorescence (n=3). Error bars denote SEM. Significance was determined by two-sided unpaired t test.

c) DNA-end resection is defective in *BLM*$^{-/-}$ cells as measured by RPA loading on chromatin in response to 1 µM CPT treatment for 1 hr in a FACS-based assay (n=3). Error bars denote SEM. Significance was determined by two-sided unpaired t test.

d) Colony survival assay showing that *BLM*$^{-/-}$ cells are hypersensitive to HU (n=3). Cells were treated with the indicated doses of HU for 24 hr. "cl." = clone. Error bars denote SEM.

e) Experimental outline of the DNA fibre assay to measure replication fork restart.

f) Quantification of the replication fork restart defect in *BLM*$^{-/-}$ cells by DNA fibre assay (n=2, one representative experiment shown). At least 100 DNA fibres were analysed per sample. Significance was determined using the two-sided Mann-Whitney *U* test.

g) RAD51 foci are not affected by BLM loss in RPE-1 cells. Cells were pulsed with 10 µM EdU for 15 min to mark sites of active DNA replication and treated where indicated with 2 mM HU for 2 hr. Scale bars, 5 µm.

h) Quantification of RAD51 foci in Cyclin A-positive cells, treated where indicated with 2 mM HU for 24 hr (n=2, one representative experiment shown). ~100 cells were analysed per sample. Significance was determined using the two-sided Mann-Whitney *U* test.

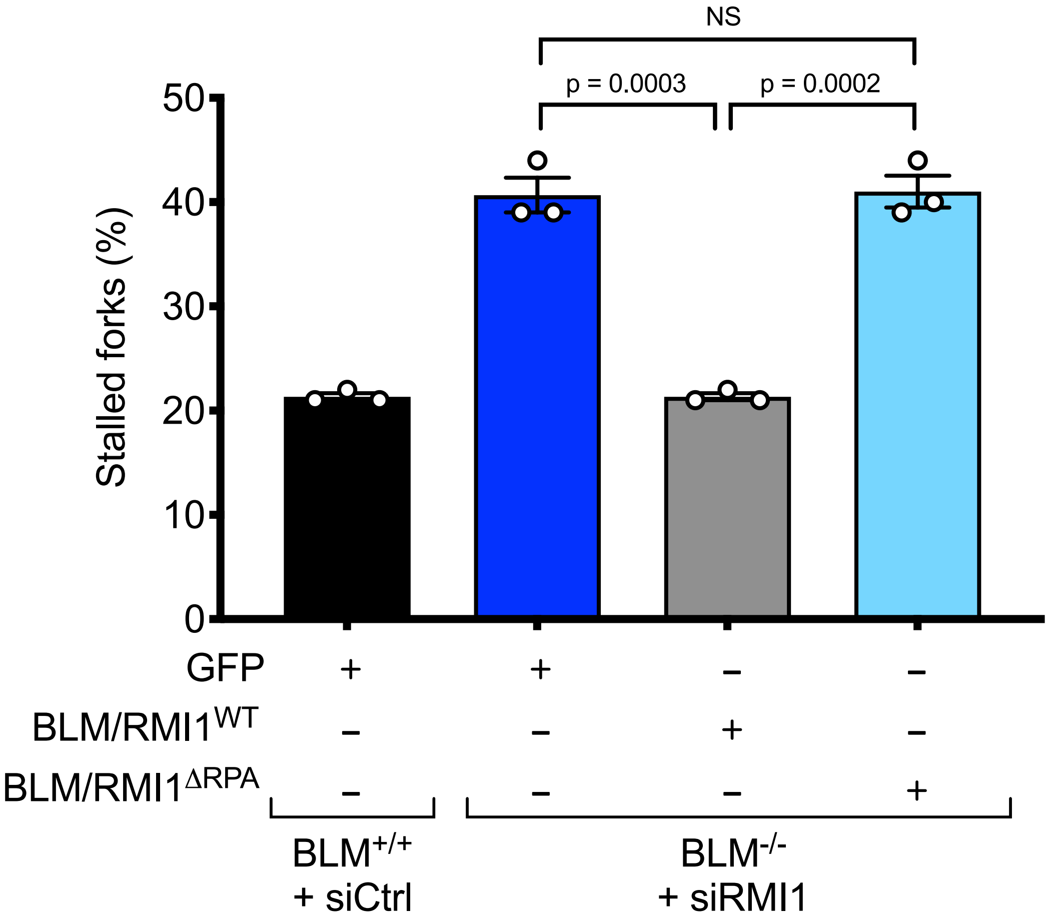

Fig. S4. RPA-binding by BLM and RMI1 suppresses increased stalled replication forks.

DNA fibre assays were carried out as in Supplementary Fig. 3e. The number of IdU-positive, CldU-negative tracts was quantified as a percentage of total fibres. At least 100 DNA fibres were analysed per sample. Significance was determined by two-sided unpaired t test (n=3, error bars denote SEM).

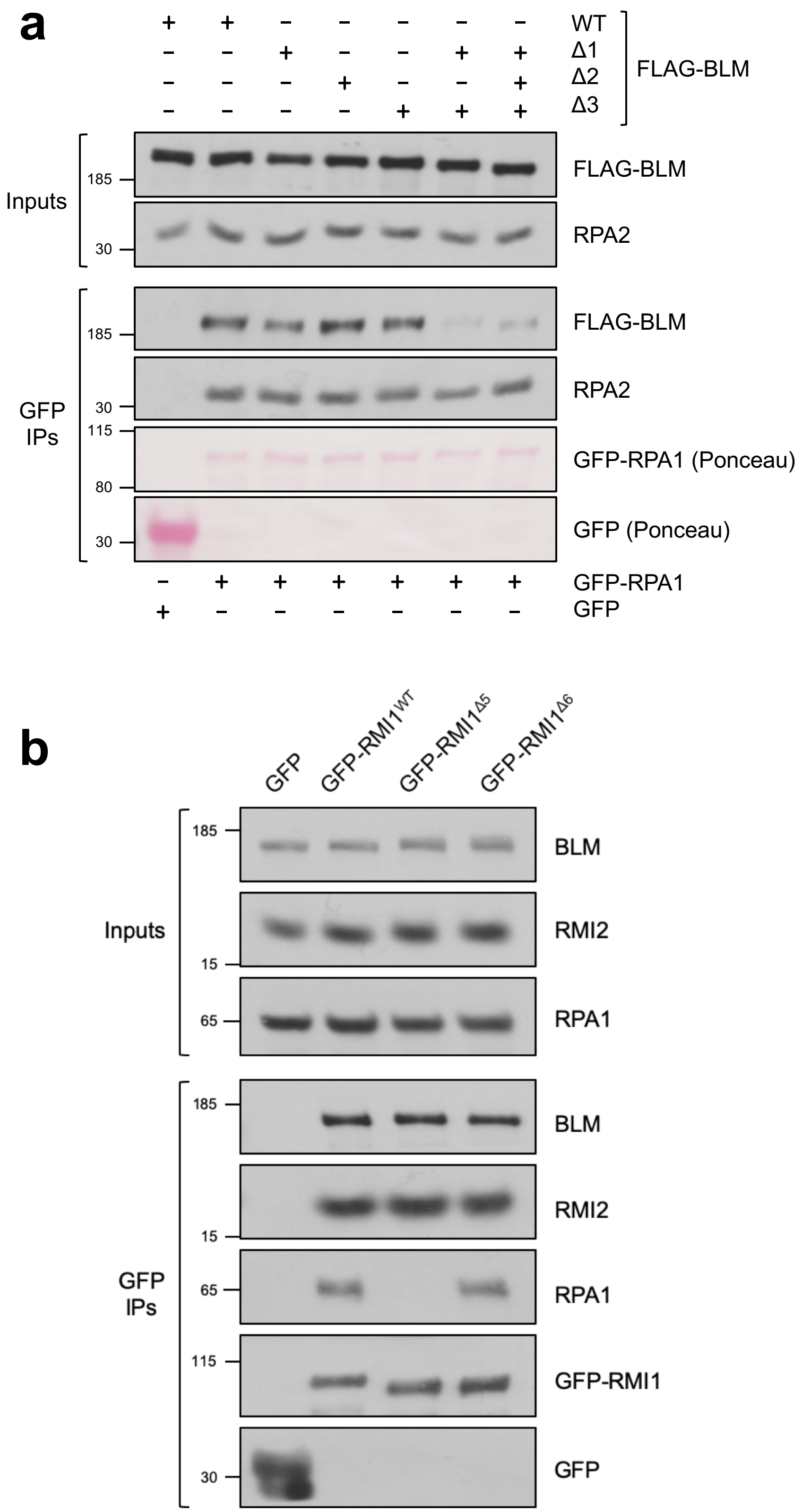

Fig. S5. BLM motif 2 and RMI1 motif 6 are not required for RPA-binding.

a) GFP-pulldowns from 293FT cells transfected with constructs expressing either GFP or GFP-RPA1, and the indicated FLAG-tagged BLM variants, showing how loss of motifs 1 and 3 but not motif 2 of BLM impacts on RPA-binding.

b) GFP-pulldowns from 293FT cells transfected with constructs expressing GFP or the indicated GFP-tagged RMI1 variants, showing that loss of motif 5 disrupts RPA-binding but motif 6 does not.

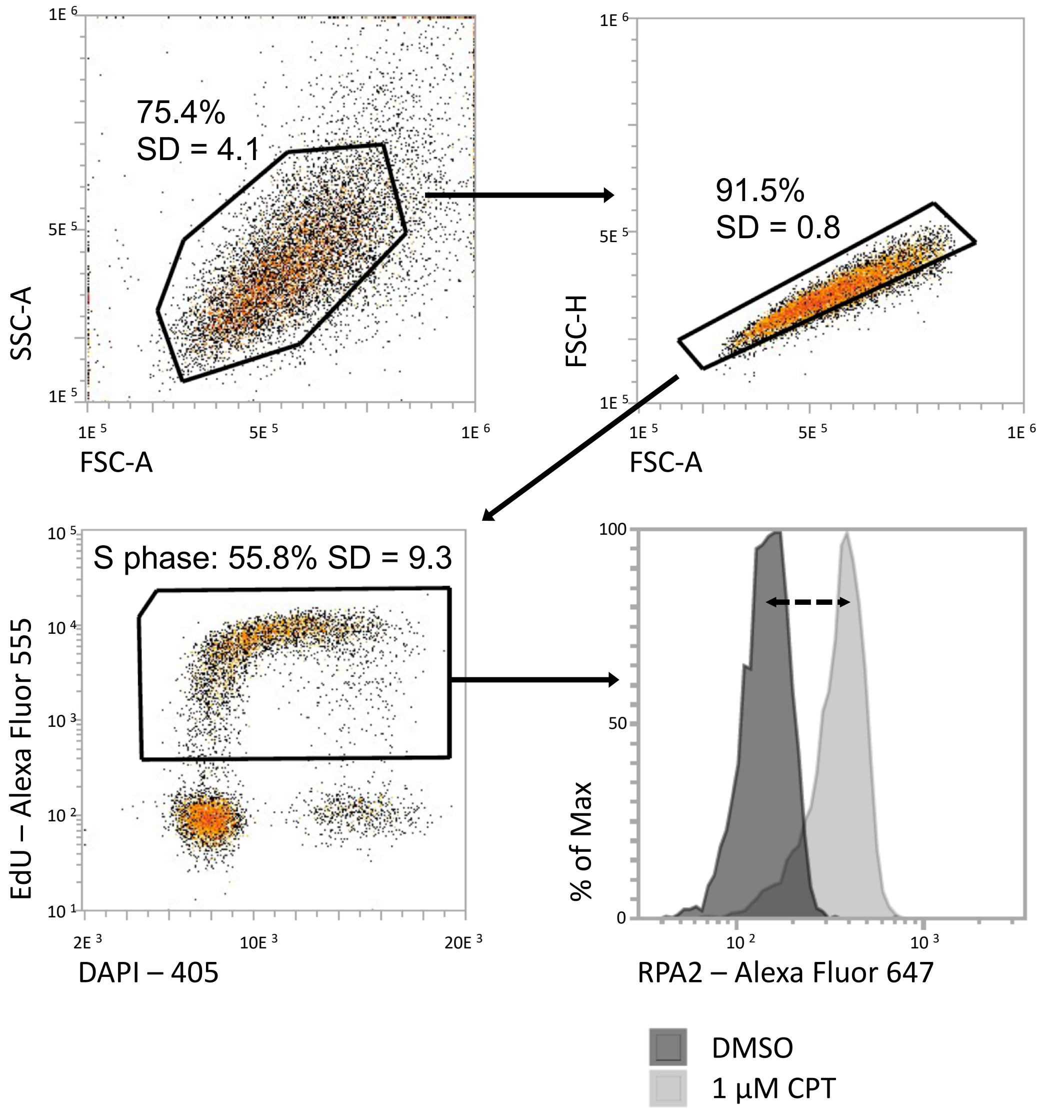

Fig. S6. Flow cytometry gating strategy.

Single RPE-1 cells were identified using Forward Scatter (FSC) and Side Scatter (SSC) and gated as indicated (top left panel, top right panel). EdU pulse-labelling was used to identify replicating cells in S phase and DAPI was used to quantify DNA content, before gating as indicated. Fluorescent marker/excitation wavelengths used are indicated on the axes (bottom left panel). Mean RPA2 intensity was calculated from the S phase population and the difference between DMSO- and CPT-treated cells found to quantify CPT-induced resection, as indicated by the dotted arrow. Fluorescent marker and excitation wavelength used are indicated on the x-axis (bottom right panel). The percentage of cells gated at each step is indicated on the panels (n = 3, SD = standard deviation).
