# Supplementary material for: The Bloom syndrome complex senses RPA-coated single-stranded DNA to restart stalled replication forks
Source: Nat Commun. 2021 Jan 26;12:585. doi: 10.1038/s41467-020-20818-5 (PMC7838300; doi:10.1038/s41467-020-20818-5)
Supplement: Supplementary file 3 — Description of Additional Supplementary Files [file 41467_2020_20818_MOESM3_ESM.pdf]

Description of Additional Supplementary Files

File Name: Supplementary Data 1-7
Description: Raw mass spectrometry data from peptide pulldowns

File Name: Supplementary Data 8
Description: BLM allele sequencing after CRISPR-Cas9 targeting

File Name: Supplementary Data 9
Description: List of primers used in this study
