# Supplementary material for: The Bloom syndrome complex senses RPA-coated single-stranded DNA to restart stalled replication forks
Source: Nat Commun. 2021 Jan 26;12:585. doi: 10.1038/s41467-020-20818-5 (PMC7838300; doi:10.1038/s41467-020-20818-5)
Supplement: Supplementary file 5 — Reporting Summary [file 41467_2020_20818_MOESM5_ESM.pdf]

nature research

Corresponding author(s): Andrew N. Blackford

Last updated by author(s): 2020/11/10

Reporting Summary

Nature Research wishes to improve the reproducibility of the work that we publish. This form provides structure for consistency and transparency in reporting. For further information on Nature Research policies, see Authors & Referees and the Editorial Policy Checklist.

Statistics

For all statistical analyses, confirm that the following items are present in the figure legend, table legend, main text, or Methods section.

n/a	Confirmed	
☐	☒	The exact sample size (*n*) for each experimental group/condition, given as a discrete number and unit of measurement
☐	☒	A statement on whether measurements were taken from distinct samples or whether the same sample was measured repeatedly
☐	☒	The statistical test(s) used AND whether they are one- or two-sided<br>*Only common tests should be described solely by name; describe more complex techniques in the Methods section.*
☐	☒	A description of all covariates tested
☐	☒	A description of any assumptions or corrections, such as tests of normality and adjustment for multiple comparisons
☐	☒	A full description of the statistical parameters including central tendency (e.g. means) or other basic estimates (e.g. regression coefficient) AND variation (e.g. standard deviation) or associated estimates of uncertainty (e.g. confidence intervals)
☐	☒	For null hypothesis testing, the test statistic (e.g. *F*, *t*, *r*) with confidence intervals, effect sizes, degrees of freedom and *P* value noted<br>*Give P values as exact values whenever suitable.*
☒	☐	For Bayesian analysis, information on the choice of priors and Markov chain Monte Carlo settings
☒	☐	For hierarchical and complex designs, identification of the appropriate level for tests and full reporting of outcomes
☒	☐	Estimates of effect sizes (e.g. Cohen's *d*, Pearson's *r*), indicating how they were calculated

Our web collection on statistics for biologists contains articles on many of the points above.

Software and code

Policy information about availability of computer code

Data collection	All images were acquired using an LSM 880 Airyscan inverted microscope (ZEISS) equipped with a 63x/1.4 NA Plan Apochromat objective and an Airyscan 32-pinhole detector unit. For Airyscan imaging, raw data were processed using Airyscan processing with Wiener Filter strength 6 using ZEN Black software version 2.1.
Data analysis	For statistical analyses, Microsoft Excel for Mac and GraphPad Prism 8 for macOS were used. Graphs were generated using GraphPad Prism 8 for macOS. Images were analysed in Fiji. Figures were prepared using Microsoft Powerpoint for Mac.

For manuscripts utilizing custom algorithms or software that are central to the research but not yet described in published literature, software must be made available to editors/reviewers. We strongly encourage code deposition in a community repository (e.g. GitHub). See the Nature Research guidelines for submitting code & software for further information.

Data

Policy information about availability of data

All manuscripts must include a data availability statement. This statement should provide the following information, where applicable:

- Accession codes, unique identifiers, or web links for publicly available datasets
- A list of figures that have associated raw data
- A description of any restrictions on data availability

The datasets generated during and/or analysed during the current study are available from the corresponding author on reasonable request. The mass spectrometry proteomics data have been deposited to the ProteomeXchange Consortium via the PRIDE partner repository with the dataset identifier PXD018322 and 10.6019/PXD018322.

Field-specific reporting

Please select the one below that is the best fit for your research. If you are not sure, read the appropriate sections before making your selection.

☒ Life sciences ☐ Behavioural & social sciences ☐ Ecological, evolutionary & environmental sciences

For a reference copy of the document with all sections, see nature.com/documents/nr-reporting-summary-flat.pdf

Life sciences study design

All studies must disclose on these points even when the disclosure is negative.

Sample size	Sample size was not predetermined
Data exclusions	No data were excluded
Replication	Most experiments were repeated at least 3 times, unless stated differently in figure legends. Sample size and number of independent experiments are stated in the figure legends.
Randomization	No randomization was applied as this study does not involve animals or human participants.
Blinding	No blinding was applied during sample collection and processing, but where indicated software was used to quantify data to avoid bias.

Reporting for specific materials, systems and methods

We require information from authors about some types of materials, experimental systems and methods used in many studies. Here, indicate whether each material, system or method listed is relevant to your study. If you are not sure if a list item applies to your research, read the appropriate section before selecting a response.

Materials & experimental systems

n/a	Involved in the study	
☐	☒	Antibodies
☐	☒	Eukaryotic cell lines
☒	☐	Palaeontology
☒	☐	Animals and other organisms
☒	☐	Human research participants
☒	☐	Clinical data

Methods

n/a	Involved in the study	
☒	☐	ChIP-seq
☐	☒	Flow cytometry
☒	☐	MRI-based neuroimaging

Antibodies

Antibodies used

Antigen (host, catalogue number, supplier, dilution for IB):
BLM (rabbit, A300-110A, Bethyl Laboratories, 1/2000)
FLAG (mouse, F1804, Sigma-Aldrich, 1/2000)
GFP (mouse, 11814460001, Roche, 1/5000)
MRE11 (rabbit, PC388, Merck, 1/500)
RMI1 (rabbit, NB100-1720, Novus Biologicals, 1/1000)
RMI2 (rabbit, NBP1-89962, Novus Biologicals, 1/4000)
RPA1 (mouse, NA13, Merck, 1/200)
RPA2 (rabbit, ab10359, Abcam, 1/10,000)
RPA3 (rabbit, HPA005708, Sigma-Aldrich, 1/1000)
TOP3A (rabbit, 14525-1-1AP, Proteintech, 1/1000)
TOPBP1 (rabbit, A300-111A, Bethyl Laboratories, 1/2000)
XPA (mouse, ab65963, Abcam, 1/2000)

Antigen (host, catalogue number, supplier, dilution for IF/FACS):
BrdU (mouse, 347580, BD Biosciences, 1/25)
BrdU (rat, ab6326, Abcam, 1/400)
Cyclin A (mouse, 611268, BD Biosciences, 1/200)
GFP (rabbit, PABG1, ChromoTek, 1/500)
γH2AX (mouse, 05-636, Merck, 1/500)
γH2AX (rabbit, 2577, Cell Signaling Technology, 1/500)
RAD51 (rabbit, 70-002, BioAcademia, 1/1000)
RIF1 (mouse, sc-515573, Santa Cruz Biotechnology, 1/50)
RPA2 (mouse, ab2175, Abcam, 1/300)
Alexa Fluor 488 goat anti-rabbit IgG (A11034, Thermo Fisher Scientific, 1/1000)

	Alexa Fluor 488 goat anti-rat IgG (A11006, Thermo Fisher Scientific, 1/500)<br>Alexa Fluor 568 donkey anti-mouse IgG (A10037, Thermo Fisher Scientific, 1/500 or 1/1000 where indicated)<br>Alexa Fluor 568 donkey anti-rabbit IgG (A10042, Thermo Fisher Scientific, 1/1000)<br>Alexa Fluor 647 goat anti-mouse IgG1 (A21240, Thermo Fisher Scientific, 1/200 or 1/1000 where indicated)
Validation	Commercially available antibodies were validated by the supplier, in previous publications and/or by us using appropriate controls where needed such as siRNA or CRISPR-Cas9 to deplete antigens.

Eukaryotic cell lines

Policy information about cell lines

Cell line source(s)	RPE-1 FRT/TR cells were obtained from Ximbio. 293FT cells were purchased from Thermo Fisher Scientific.
Authentication	All cells were originally obtained and authenticated by the indicated supplier.
Mycoplasma contamination	All cell lines were routinely tested to confirm mycoplasma-free status using a LookOut Mycoplasma PCR Detection Kit (Sigma-Aldrich).
Commonly misidentified lines<br>(See ICLAC register)	No commonly misidentified cell lines were used.

Flow Cytometry

Plots

Confirm that:

☒ The axis labels state the marker and fluorochrome used (e.g. CD4-FITC).

☒ The axis scales are clearly visible. Include numbers along axes only for bottom left plot of group (a 'group' is an analysis of identical markers).

☒ All plots are contour plots with outliers or pseudocolor plots.

☒ A numerical value for number of cells or percentage (with statistics) is provided.

Methodology

Sample preparation	See Methods section for further details.
Instrument	Life Technologies Attune NxT analyser
Software	Attune NxT Software v3.2.1
Cell population abundance	N/A
Gating strategy	See Supplementary fig. 6 for details of gating strategy. 20,000 cells were analyzed per sample.

☒ Tick this box to confirm that a figure exemplifying the gating strategy is provided in the Supplementary Information.
